# Electroosmosis and Solute Diffusion Transport of Maxwell Fluid Through a Polyelectrolyte-Grafted Microchannel with Modulated Charged Surfaces

**DOI:** 10.3390/mi17050613

**Published:** 2026-05-16

**Authors:** Yin Shang, Fengqin Li, Chunhong Yang

**Affiliations:** School of Mathematical Sciences, Inner Mongolia University, Hohhot 010030, China; sy20016020@163.com (Y.S.); skyyang325@163.com (C.Y.)

**Keywords:** polyelectrolyte-grafted microchannel, modulated charged surface, Maxwell fluid, electroosmotic flow, solute diffusion transport

## Abstract

This study investigates the time-periodic electroosmotic flow and solute transport of Maxwell fluid in a parallel microchannel with modulated surface charges. The Poisson–Boltzmann equation and the linearized momentum equations are solved using a superposition-based analytical approach. The influences of oscillation intensity, fluid elasticity, and electrokinetic parameters on the velocity and concentration distributions are examined. The results show that wall-potential modulation combined with a time-periodic electric field generates recirculating motion and oscillatory velocity patterns. Moderate oscillation strengthens both flow and solute transport, whereas stronger oscillation weakens transport efficiency. This work provides a quantitative analysis the interplay between oscillatory electroosmotic flow and solute transport in Maxwell fluid and clarifies the role of oscillation strength in controlling solute dispersion.

## 1. Introduction

Microfluidic and nanofluidic systems have attracted significant interest due to their broad applications in biomedical diagnostics, molecular separation, and seawater desalination [1,2,3,4,5]. In recent years, studies have concentrated mainly on fundamental topics such as fluid transport, mixing behavior, and heat transfer processes within microchannels, where the flow can be driven by various forces such as pressure gradients, electric and magnetic fields, and acoustic waves [6,7,8]. In addition, extensive efforts have been devoted to examining the influence of channel geometry, surface characteristics, and external stimuli, with the aim of enhancing device performance and achieving precise control of microscale fluid transport [9,10,11,12].

Electroosmotic flow (EOF) is generated when an external electric field is imposed on a microchannel filled with an electrolyte solution [13]. The charged walls of the channel attract oppositely charged ions from the solution, leading to the formation of a thin interfacial region referred to as the electric double layer (EDL) [14]. Under the action of the electric field, the mobile ions within the EDL move along the field direction and entrain the surrounding fluid, thereby inducing bulk fluid motion without the need for mechanical driving components. Due to its simple configuration, stable flow characteristics, and ease of control, EOF has been widely applied in biological analysis, chemical processing, and medical diagnostics [15,16]. Although conventional EOF models assume rigid channel walls, many practical micro- and nanofluidic systems feature surface modifications that markedly influence electrokinetic behavior. Polyelectrolyte-grafted (PE-grafted) channels have attracted considerable interest due to their distinctive interfacial characteristics and adjustable electrohydrodynamic behavior.

The concept of polyelectrolyte-grafted (PE-grafted) microchannels and nanochannels has attracted considerable interest in both theoretical research and practical applications [17,18,19,20,21]. In these soft channels, the inner surfaces are coated with brush-like layers of charged polymer chains, forming a polyelectrolyte layer (PEL). A PEL typically has a thickness less than half the channel height, allowing electrolyte ions from the bulk solution to penetrate the layer. This forms a region that behaves as a semi-permeable medium containing both mobile ions and immobile fixed charges. Numerous studies have investigated electrokinetic and transport phenomena in PE-grafted systems under various conditions. For example, Li et al. [22] analyzed AC EOF in nanochannels with brush-like PELs. They reported that the PEL enhances EOF velocity while suppressing velocity oscillations near the PEL-electrolyte interface at high oscillating Reynolds numbers. Wang and Li [23] investigated EOF and heat transfer of a Newtonian fluid in PEL-coated microchannels with modulated surface charges. They showed that the PEL significantly alters the flow field and temperature distribution due to the combined effects of surface modulation and Joule heating. Li and Jian [24] examined solute dispersion in AC-driven EOF with interfacial slip and found that thicker PELs lead to higher fluid velocity and enhanced dispersion. Zhang et al. [25] investigated electrokinetic energy conversion in PE-grafted nanochannels under periodic pressure and magnetic fields, emphasizing the role of couple-stress effects in the PEL.

Electroosmotic flow (EOF) can be regulated through various strategies. In addition to internal control methods such as surface charge modulation via polyelectrolyte grafting or chemical coatings, external electric fields, particularly radial electric fields, have also been employed as an effective way to manipulate EOF [26,27]. Recently, numerous studies have investigated the effects of modulated charged surfaces on microfluidic and nanofluidic flows. In practical microfluidic and bio-interfacial systems, surface charges are seldom uniform. They often exhibit spatial variations as a result of chemical heterogeneity, ion adsorption, or intentional patterning via microfabrication techniques. Such charge modulation can significantly influence the EOF characteristics and the local ion distribution near the channel walls [28,29,30,31,32]. Sun et al. [33] analytically investigated the time-periodic electroosmotic flow of viscoelastic fluids in microchannels and demonstrated that modulated surface charges can enhance the velocity, volumetric flow rate, and mixing performance. Qing, Wang and Li [34] investigated the EOF and the solute diffusion transport of a Newtonian fluid in a modulated microchannel under combined pressure and AC electric fields, showing that the velocity amplitude increases with modulation potential. Mandal and Ghosh [35] studied the EOF of two immiscible fluids confined within narrow channels under axially modulated surface charges. Yang et al. [36] examined time-periodic electroosmotic flow in a pH-regulated nanochannel, revealing that the flow velocity decreases as the salt concentration increases, but rises as the pH deviates further from the isoelectric point.

Generally, the electrokinetic behavior of modulated charged surfaces has been investigated based on the Newtonian fluid assumption. However, many biological and polymeric fluids encountered in microfluidic systems exhibit non-Newtonian rheological properties, which cannot be adequately described by Newtonian models. To account for these complex behaviors, a variety of non-Newtonian fluid models have been proposed, including power-law fluids, Oldroyd-B fluids, and Maxwell-type viscoelastic fluids [37,38,39,40,41]. Among these models, the Maxwell fluid is particularly useful for describing viscoelastic effects with a single relaxation time, and it has been widely employed to capture the elastic response of biological fluids such as blood under certain flow conditions [42]. Bandopadhyay and Ghosh [43] studied EOF of Maxwell fluid under time-periodic electric fields, highlighting the coupling between interfacial electrodynamics and rheology. Liu, Jian, and Yang [44] investigated the electrokinetic flow of the Maxwell fluid and showed that both the velocity and flow rate depend strongly on the electrokinetic width, oscillating Reynolds number, and the fluid relaxation time. Zakaria et al. [45] examined the stability of a modulated electrokinetic flow of the Maxwell fluid and found that the Reynolds number destabilizes the flow, whereas the relaxation time plays a stabilizing role.

Solute diffusion transport is a molecular-scale process in which particles move from regions of higher concentration to regions of lower concentration driven by concentration gradients. In contrast, convective solute diffusion transport occurs when the fluid itself is in motion. In this case, the solutes are carried along with the flow, enabling transport across space through the bulk movement of the fluid [46,47,48,49]. Patmonoaji et al. [50] developed a solute diffusion transport model based on Sherwood, Reynolds, and Schmidt numbers. Roy and Bhattacharjee [51] studied solute diffusion transport in a polymer-grafted charged nanochannel under combined electroosmotic and pressure-driven flow. Lutsenko et al. [52] showed that the velocity amplitude mainly affects solute diffusion transport, whereas the oscillation frequency has little impact.

To the best of the authors’ knowledge, no studies have examined the EOF and solute diffusion transport of Maxwell fluids in a parallel-plate microchannel under the combined effects of an AC electric field and modulated surface charges. Therefore, the present study investigates this system. The modified Navier–Stokes equations for Maxwell fluids are solved by asymptotic expansion, while the solute concentration equation is computed numerically using a finite-difference perturbation approach. The influences of surface charge modulation, AC electric fields, and Maxwell fluid rheology on the potential, velocity and solute diffusion transport are then examined in detail.

## 2. Mathematics Methods

**Figure 1:**
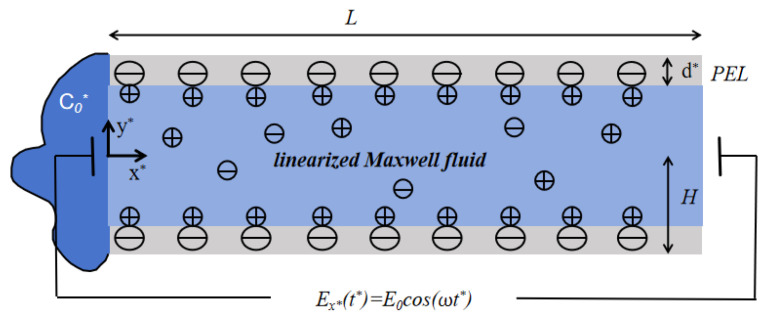
The schematic of the mathematical model of the cross-section for the polyelectrolyte-grafted microchannel. $E_{0}$ is amplitude of electric field and ω is oscillation frequency.

The mathematical model of the cross-section for the polyelectrolyte-grafted microchannel is shown in Figure 1. The height of the parallel plate is 2H and the length is *L*(L≫2H). The $y^{*}$-axis is defined as the normal direction of the wall, while the $x^{*}$-axis is taken along the tangential direction. The bottom plate is located at $y^{*}=-H$, and the top plate is located at $y^{*}=H$. The applied electric field acts on the fluid along the $x^{*}$ direction. The thickness of the PEL is $d^{*}$, and $d^{*}$ is much smaller than *H*. In polyelectrolyte-grafted microchannels, the regions $-H\leq y^{*}\leq -H+d^{*}$ and $H-d^{*}\leq y^{*}\leq H$ represent the PEL within the microchannel. The fluid inside the microchannel is regarded as a linearized Maxwell fluid. The dynamic viscosity of the fluid is expressed as η, the density as ρ, and the relaxation time as $t_{m}$. The permittivity of the fluid is indicated by ε. Due to the symmetry of the channel and the electrolyte solution, only half of the channel ($0\leq y^{*}\leq H$) is considered.

When the surface charge is modulated, the zeta potentials on the upper and lower plates can be expressed as follows:

(1) $${\left.\psi^{*}\right|}_{y^{*}=\pm H}=\xi_{0}^{*}\left[1+\alpha \cos\left(m^{*}x^{*}\right)\right],$$

here, $\xi_{0}^{*}$ is the modulated potential amplitude, α is the modulation parameter, and $m^{*}$ is the mode frequency.

### 2.1. EDL Potential Distribution

In polyelectrolyte-grafted microchannels, assuming they are fully filled with symmetric electrolyte solutions, the EDL potential distribution can be described by the following Poisson–Boltzmann equation [23]:

(2) $$\frac{\partial^{2}\psi^{*}}{\partial {x^{*}}^{2}}+\frac{\partial^{2}\psi^{*}}{\partial {y^{*}}^{2}}=-\frac{\rho_{e}}{\varepsilon },0\leq y^{*}\leq H-d^{*}$$

(3) $$\frac{\partial^{2}\psi^{*}}{\partial {x^{*}}^{2}}+\frac{\partial^{2}\psi^{*}}{\partial {y^{*}}^{2}}=-\frac{\rho_{e}+ZeN}{\varepsilon },H-d^{*}\leq y^{*}\leq H$$

(4) $$\rho_{e}=-2ezn_{0}sinh\left(ez\psi^{*}\right)/\left(K_{B}T_{0}\right)$$

Here, ε denotes the electric permittivity of the electrolyte solution, which is assumed to be identical both inside and outside the PEL. The parameter $\rho_{e}$ represents the net charge density of the electrolyte, *e* is the elementary charge, and $n_{0}$ is the bulk ion concentration in the solute, *z* is the absolute value of the ionic valence, $K_{B}$ is the Boltzmann constant, and *Z* and *N* are the valence and the concentration of ionic numbers of fixed charge ions in the PEL, respectively.

In addition, if the wall potential is sufficiently small (far less than 25 mV), the electrostatic potential energy is much smaller than the thermal energy of the ions, that is $\left|\frac{ez\psi^{*}}{K_{B}T_{0}}\right|\ll 1$. Then Debye–Hückel linearized approximation can be applied to Equation (4) by using the expression $\sinh\;\left(\frac{ez\psi^{*}}{K_{B}T_{0}}\right)\approx \frac{ez\psi^{*}}{K_{B}T_{0}}$. The above equation can be rewritten as follows:

(5) $$\frac{\partial^{2}\psi^{*}}{\partial {x^{*}}^{2}}+\frac{\partial^{2}\psi^{*}}{\partial {y^{*}}^{2}}=\frac{\psi^{*}}{{\left(\lambda^{*}\right)}^{2}},0\leq y^{*}\leq H-d^{*}$$

(6) $$\frac{\partial^{2}\psi^{*}}{\partial {x^{*}}^{2}}+\frac{\partial^{2}\psi^{*}}{\partial {y^{*}}^{2}}=\frac{\psi^{*}}{{\left(\lambda^{*}\right)}^{2}}-\frac{\psi^{*}}{{\left(\lambda_{F}^{*}\right)}^{2}},H-d^{*}\leq y^{*}\leq H$$

where $\lambda^{*}={\left[\frac{\varepsilon K_{B}T_{0}}{2n_{\infty }e^{2}z^{2}}\right]}^{1/2}$ is the thickness of the EDL, and $\lambda_{F}^{*}={\left[\frac{\varepsilon K_{B}T_{0}}{Ze^{2}zN}\right]}^{1/2}$ is the equivalent EDL thickness in PEL. The boundary conditions are

(7) $$\left\{\begin{array}{c} \;\psi^{*}{|}_{y^{*}=\pm H}=\xi_{0}^{*}\left[1+\alpha \cos\left(m^{*}x^{*}\right)\right],\\ \;{\left.\frac{\partial \psi^{*}}{\partial y^{*}}\right|}_{y^{*}=0}=0,\\ \;\psi^{*}{{|}_{y^{*}={\left(H-d^{*}\right)}^{+}}=\psi^{*}|}_{y^{*}={\left(H-d^{*}\right)}^{-}},\\ \;{\left.\frac{\partial \psi^{*}}{\partial y^{*}}\right|}_{y^{*}={\left(H-d^{*}\right)}^{+}}={\left.\frac{\partial \psi^{*}}{\partial y^{*}}\right|}_{y^{*}={\left(H-d^{*}\right)}^{-}}. \end{array}\right.$$

Equation (7) specifies the boundary conditions for the electric potential, including the prescribed wall potential, symmetric about the $x^{*}$ axis and the continuity of both the electric potential and the electric field at the interface between the microchannel layer and the electrolyte solution.

The following dimensionless variations are introduced: $x=\frac{x^{*}}{L},\;y=\frac{y^{*}}{H},\;m=m^{*}H,$ $\psi =\frac{\psi^{*}}{\psi_{s}},\;d=\frac{d^{*}}{H},\;\lambda =\frac{\lambda^{*}}{H},\;\lambda_{F}=\frac{\lambda_{F}^{*}}{H},\;\xi_{0}=\frac{\xi^{*}}{\psi_{S}},\;\psi_{s}=\frac{K_{B}T_{0}}{ez}$.

Then, Equations (5) and (6) and the corresponding boundary conditions Equation (7) can be modified as follows:

(8) $$\beta^{2}\frac{\partial^{2}\psi }{\partial x^{2}}+\frac{\partial^{2}\psi }{\partial y^{2}}=\frac{\psi }{\lambda^{2}},0\leq y\leq 1-d$$

(9) $$\beta^{2}\frac{\partial^{2}\psi }{\partial x^{2}}+\frac{\partial^{2}\psi }{\partial y^{2}}=\frac{\psi }{\lambda^{2}}-\frac{\psi }{\lambda_{F}^{2}},1-d\leq y\leq 1$$

(10) $$\left\{\begin{array}{c} \;{\left.\psi \right|}_{y=\pm 1}=\xi_{0}\left(1+\alpha \cos\left(mx\right)\right),\\ \;{\left.\frac{\partial \psi }{\partial y}\right|}_{y=0}=0,\\ {\;\psi |}_{y={\left(1-d\right)}^{+}}{=\psi |}_{y={\left(1-d\right)}^{-}},\\ \;{\left.\frac{\partial \psi }{\partial y}\right|}_{y={\left(1-d\right)}^{+}}={\left.\frac{\partial \psi }{\partial y}\right|}_{y={\left(1-d\right)}^{-}}. \end{array}\right.$$

In the above equation, β=H/L is the dimensionless ratio of the half-height to the length of the microchannel. To maintain the continuity of potential within the microchannel, it is assumed that the thickness of PEL *d* is less than the thickness of EDL λ. Since Equations (8) and (9) are linear, their solutions can be expressed according to the superposition principle as follows:

(11) $$\psi_{1}\left(x,y\right)=f_{1}\left(y\right)+f_{2}\left(y\right)cos\left(mx\right),0\leq y\leq 1-d$$

(12) $$\psi_{2}\left(x,y\right)=f_{3}\left(y\right)+f_{4}\left(y\right)cos\left(mx\right),1-d\leq y\leq 1$$

Here, the function $f_{i}\left(y\right)\left(i=1,2,3,4\right)$ satisfies the boundary conditions, respectively. Finally, we can derive it as:

(13) $$f_{1}\left(y\right)=\left[-\frac{\sinh(D)\sinh(1/\lambda )-1}{\Lambda^{2}\cosh\left(1/\lambda \right)}+\frac{\cosh(D)}{\Lambda^{2}}\right]\cosh\left(y/\lambda \right),$$

(14) $$f_{2}\left(y\right)=\frac{\alpha \xi_{0}}{\cosh(M)}\cosh\left(My\right),$$

(15) $$f_{3}\left(y\right)=\frac{\sinh(D)}{\Lambda^{2}}\sinh\left(y/\lambda \right)+\left[\frac{\Lambda^{2}\xi_{0}-\sinh\left(D\right)\sinh\left(1/\lambda \right)-1}{\Lambda^{2}\cosh\left(1/\lambda \right)}\right]\cosh\left(y/\lambda \right)+\frac{1}{\Lambda^{2}},$$

(16) $$f_{4}\left(y\right)=\frac{\alpha \xi_{0}}{\cosh(M)}\cosh\left(My\right),$$

among them, $M^{2}=m^{2}\beta^{2}+\frac{1}{\lambda^{2}}$, $D=\frac{1-d}{\lambda }$ and $\Lambda =\frac{\lambda_{F}}{\lambda }$ represent the ratio of the equivalent EDL thickness within PEL to the EDL thickness within the microchannel.

### 2.2. Electroosmotic Flow

We assume that the structure of the polyelectrolyte brushes within the PEL remains unchanged under flow. We consider the drag coefficient within the PEL constant. The continuity equations of the inner and outer regions of PEL under the action of pressure gradient, electric field force, resistance, and the modified Navier–Stokes equation are as follows [23]:

(17) $$\nabla \cdot u^{*}=0,$$

(18) $$\rho \frac{Du^{*}}{Dt^{*}}=-\nabla P^{*}+\nabla \cdot \tau +F,0\leq y^{*}\leq H-d^{*},$$

(19) $$\rho \frac{Du^{*}}{Dt^{*}}=-\nabla P^{*}+\nabla \cdot \tau +F-\mu_{c}u^{*},H-d^{*}\leq y^{*}\leq H,$$

where $u^{*}=\left(u^{*},v^{*},0\right)$ represents the velocity of the fluid, ρ is the density of the fluid, $P^{*}$ is the pressure, μ is the dynamic viscosity of the fluid, $F=\rho_{e}E_{x^{*}}\left(t^{*}\right)$ is the force of the electric field in the fluid, and the drag force generated by the brush in PEL is expressed as $\mu_{c}u^{*}$, where $\mu_{c}$ is the drag coefficient in PEL.

The fluid considered here is a linearized Maxwell fluid and its constitutive behavior can be mathematically expressed as follows:

(20) $$t_{m}\frac{\partial \tau }{\partial t^{*}}=\eta \left(\nabla u+{\left(\nabla u\right)}^{T}\right)-\tau ,$$

where $t_{m}$ is the relaxation time scale, τ is the deviatoric stress tensor, and u the velocity vector.

The boundary conditions satisfying the above equations are the following:

(21) $$\left\{\begin{array}{c} \;u^{*}{{|}_{y^{*}=H}=0,v^{*}|}_{y^{*}=H}=0,\\ \;{\left.\frac{\partial u^{*}}{\partial y^{*}}\right|}_{y^{*}=0}=0,{\left.\frac{\partial v^{*}}{\partial y^{*}}\right|}_{y^{*}=0}=0,\\ \;u^{*}{|}_{y^{*}={\left(H-d^{*}\right)}^{+}}=u^{*}{|}_{y^{*}={\left(H-d^{*}\right)}^{-}},v^{*}{{|}_{y^{*}={\left(H-d^{*}\right)}^{+}}=v^{*}|}_{y^{*}={\left(H-d^{*}\right)}^{-}},\\ \;\tau_{yx}^{*}{{|}_{y^{*}={\left(H-d^{*}\right)}^{+}}=\tau_{yx}^{*}|}_{y^{*}={\left(H-d^{*}\right)}^{-}} \end{array}\right.$$

Equation (21) imposes no-slip and no-permeation on the top wall, enforces velocity symmetry on the $x^{*}$-axis, and ensures continuity of velocity and shear stress at the PEL–electrolyte interface.

To solve the system, note that under a time-periodic electric field, the variables in Equations (17)–(19) can be assumed to take the form:

(22) $$\xi =ℜe\left(\tilde{\xi }\left(x,y\right)exp\left(i\omega t^{*}\right)\right),E_{x^{*}}\left(t^{*}\right)=ℜe\left(E_{0}exp\left(i\omega t^{*}\right)\right)$$

Here, ξ represents variables such as $u^{*}$, $v^{*}$, $P^{*}$, etc. ℜe denotes the real part.

Substituting the time-periodic form given by Equation (22) into the Maxwell constitutive Equation (20), we obtain

(23) $$t_{m}\frac{\partial }{\partial t^{*}}\left[\tilde{\tau }e^{i\omega t^{*}}\right]=\eta \left(\nabla {\tilde{u}}^{*}e^{i\omega t^{*}}+{\left(\nabla {\tilde{u}}^{*}e^{i\omega t^{*}}\right)}^{T}\right)-\tilde{\tau }e^{i\omega t^{*}}.$$

Therefore, the stress tensor can be expressed as

(24) $$\tilde{\tau }=\frac{\eta }{1+i\omega t_{m}}\left(\nabla {\tilde{u}}^{*}+{\left(\nabla {\tilde{u}}^{*}\right)}^{T}\right).$$

For an incompressible fluid with $\nabla \cdot \tilde{u}=0$, it follows that

(25) $$\nabla \cdot \tilde{\tau }=\frac{\eta }{1+i\omega t_{m}}\nabla^{2}\tilde{u}.$$

In Equations (18) and (19), the advection terms are neglected because the fluid is incompressible. Substituting the form Equation (22) into Equations (17)–(20), we obtain the governing equations as follows (where ${\tilde{u}}^{*}$, ${\tilde{v}}^{*}$ and ${\tilde{P}}^{*}$ denote the steady-state components of the *x*-velocity, *y*-velocity and pressure):

For the electrolyte layer: $0\leq y\leq H-d^{*}$

(26a) $$\frac{\partial {\tilde{u}}^{*}}{\partial x^{*}}+\frac{\partial {\tilde{v}}^{*}}{\partial y^{*}}=0,$$

(26b) $$-i\omega \rho \tilde{u^{*}}=-\frac{\partial {\tilde{P}}^{*}}{\partial x^{*}}+\frac{\eta }{\varphi }\nabla^{2}{\tilde{u}}^{*}-\frac{\psi^{*}}{{\left(\lambda^{*}\right)}^{2}}\varepsilon E_{0},$$

(26c) $$-i\omega \rho \tilde{v^{*}}=-\frac{\partial {\tilde{P}}^{*}}{\partial y^{*}}+\frac{\eta }{\varphi }\nabla^{2}{\tilde{v}}^{*},$$

For the PEL: $H-d^{*}\leq y\leq H$

(27a) $$\frac{\partial {\tilde{u}}^{*}}{\partial x^{*}}+\frac{\partial {\tilde{v}}^{*}}{\partial y^{*}}=0,$$

(27b) $$-i\omega \rho \tilde{u^{*}}=-\frac{\partial {\tilde{P}}^{*}}{\partial x^{*}}+\frac{\eta }{\varphi }\nabla^{2}{\tilde{u}}^{*}-\frac{\psi^{*}}{{\left(\lambda^{*}\right)}^{2}}\varepsilon E_{0}-\mu_{c}{\tilde{u}}^{*},$$

(27c) $$-i\omega \rho \tilde{v^{*}}=-\frac{\partial {\tilde{P}}^{*}}{\partial y^{*}}+\frac{\eta }{\varphi }\nabla^{2}{\tilde{v}}^{*}-\mu_{c}{\tilde{v}}^{*},$$

We introduce the following dimensionless variables: $\tilde{u}=\frac{{\tilde{u}}^{*}}{u_{ref}},\tilde{v}=\frac{{\tilde{v}}^{*}L}{u_{ref}H},P=\frac{{\tilde{P}}^{*}}{P_{ref}},$$u_{ref}=\frac{\psi_{s}\varepsilon E_{0}}{\eta },P_{ref}=\frac{\eta u_{ref}L}{H^{2}},De=\omega t_{m},Re_{\omega }=\frac{\rho \omega H^{2}}{\eta }$.

Here, φ=1+iDe, and $De=\omega t_{m}$ is called the Deborah number, which is defined as the ratio of relaxation time $t_{m}$ of the fluid to oscillating time 1/ω of electric field. $Re_{\omega }$ means oscillating Reynolds number.

Equations (26a)–(27c) and the corresponding boundary conditions become:

For the electrolyte layer: 0≤y≤1−d

(28a) $$\frac{\partial \tilde{u}}{\partial x}+\frac{\partial \tilde{v}}{\partial y}=0,$$

(28b) $$\beta^{2}\frac{\partial^{2}\tilde{u}}{\partial x^{2}}+\frac{\partial^{2}\tilde{u}}{\partial y^{2}}-iRe_{\omega }\varphi \tilde{u}-\varphi \frac{\partial P}{\partial x}=\varphi \frac{\psi_{1}}{\lambda^{2}},$$

(28c) $$\beta^{4}\frac{\partial^{2}\tilde{v}}{\partial x^{2}}+\beta^{2}\frac{\partial^{2}\tilde{v}}{\partial y^{2}}-i\beta^{2}Re_{\omega }\varphi \tilde{v}-\varphi \frac{\partial P}{\partial y}=0,$$

For the PEL: 1−d≤y≤1

(29a) $$\frac{\partial \tilde{u}}{\partial x}+\frac{\partial \tilde{v}}{\partial y}=0,$$

(29b) $$\beta^{2}\frac{\partial^{2}\tilde{u}}{\partial x^{2}}+\frac{\partial^{2}\tilde{u}}{\partial y^{2}}-iRe_{\omega }\varphi \tilde{u}-{\alpha_{0}}^{2}\varphi \tilde{u}-\varphi \frac{\partial P}{\partial x}=\varphi \frac{\psi_{2}}{\lambda^{2}},$$

(29c) $$\beta^{4}\frac{\partial^{2}\tilde{v}}{\partial x^{2}}+\beta^{2}\frac{\partial^{2}\tilde{v}}{\partial y^{2}}-i\beta^{2}Re_{\omega }\varphi \tilde{v}-{\alpha_{0}}^{2}\beta^{2}\varphi \tilde{v}-\varphi \frac{\partial P}{\partial y}=0,$$

Here, $\alpha_{0}^{2}=\frac{\mu_{c}H^{2}}{\eta }$ is the dimensionless resistance parameter within the PEL and it is assumed to be constant.

The boundary conditions are as follows:

(30) $$\left\{\begin{array}{c} \;\tilde{u}{{|}_{y=1}=0,\tilde{v}|}_{y=1}=0,\\ \;{\left.\frac{\partial \tilde{u}}{\partial y}\right|}_{y=0}=0,{\left.\frac{\partial \tilde{v}}{\partial y}\right|}_{y=0}=0,\\ \;\tilde{u}{|}_{y={\left(1-d\right)}^{+}}=\tilde{u}{|}_{y={\left(1-d\right)}^{-}},\tilde{v}{{|}_{y={\left(1-d\right)}^{+}}=\tilde{v}|}_{y={\left(1-d\right)}^{-}},\\ \;{\left.\frac{\partial \tilde{u}}{\partial y}\right|}_{y={\left(1-d\right)}^{+}}={\left.\frac{\partial \tilde{u}}{\partial y}\right|}_{y={\left(1-d\right)}^{-}},{\left.\frac{\partial \tilde{v}}{\partial y}\right|}_{y={\left(1-d\right)}^{+}}={\left.\frac{\partial \tilde{v}}{\partial y}\right|}_{y={\left(1-d\right)}^{-}}, \end{array}\right.$$

To solve the above equations, the stream function ϕ(x,y) is defined according to Equation (28a) as follows:

(31) $$\tilde{u}=\frac{\partial \phi }{\partial y},\tilde{v}=-\frac{\partial \phi }{\partial x}$$

Combining Equations (28b), (28c) and Equations (29b), (29c) to eliminate pressure *P*, the equations for the stream function ϕ(x,y) can be obtained.

For the electrolyte layer: 0≤y≤1−d

(32) $$\beta^{4}\frac{\partial^{4}\phi }{\partial x^{4}}+\frac{\partial^{4}\phi }{\partial y^{4}}+2\beta^{2}\frac{\partial^{4}\phi }{\partial x^{2}\partial y^{2}}-i\beta^{2}Re_{\omega }\varphi \frac{\partial^{2}\phi }{\partial x^{2}}-iRe_{\omega }\varphi \frac{\partial^{2}\phi }{\partial y^{2}}=\frac{\varphi }{\lambda^{2}}\frac{\partial \psi_{1}}{\partial y}$$

For the PEL: 1−d≤y≤1

(33) $$\beta^{4}\frac{\partial^{4}\phi }{\partial x^{4}}+\frac{\partial^{4}\phi }{\partial y^{4}}+2\beta^{2}\frac{\partial^{4}\phi }{\partial x^{2}\partial y^{2}}-i\beta^{2}Re_{\omega }\varphi \frac{\partial^{2}\phi }{\partial x^{2}}-iRe_{\omega }\varphi \frac{\partial^{2}\phi }{\partial y^{2}}-{\alpha_{0}}^{2}\varphi \left(\beta^{2}\frac{\partial^{2}\phi }{\partial x^{2}}+\frac{\partial^{2}\phi }{\partial y^{2}}\right)=\frac{\varphi }{\lambda^{2}}\frac{\partial \psi_{2}}{\partial y}$$

For the electrolyte layer: 0≤y≤1−d, stream function $\phi_{1}\left(x,y\right)$ can be set as:

(34) $$\phi_{1}\left(x,y\right)=g_{1}\left(y\right)+g_{2}\left(y\right)cos\left(mx\right)$$

For the PEL: 1−d≤y≤1, the stream function $\phi_{2}\left(x,y\right)$ can be set as:

(35) $$\phi_{2}\left(x,y\right)=g_{3}\left(y\right)+g_{4}\left(y\right)cos\left(mx\right)$$

where $g_{i}\left(y\right)\left(i=1,2,3,4\right)$ can be calculated as:

(36) $$g_{1}\left(y\right)=C_{11}+C_{12}y+C_{13}\exp\left(\lambda_{11}y\right)+C_{14}\exp\left(\lambda_{12}y\right)+A_{1}sinh\left(y/\lambda \right),$$

(37) $$g_{2}\left(y\right)=C_{21}\exp\left(\lambda_{21}y\right)+C_{22}\exp\left(\lambda_{22}y\right)+C_{23}\exp\left(\lambda_{23}y\right)+C_{24}\exp\left(\lambda_{24}y\right)+A_{2}sinh\left(My\right),$$

(38) $$g_{3}\left(y\right)=C_{31}+C_{32}y+C_{33}\exp\left(\lambda_{33}y\right)+C_{34}\exp\left(\lambda_{34}y\right)+A_{31}sinh\left(y/\lambda \right)+A_{32}cosh\left(y/\lambda \right),$$

(39) $$g_{4}\left(y\right)=C_{41}\exp\left(\lambda_{41}y\right)+C_{42}\exp\left(\lambda_{42}y\right)+C_{43}\exp\left(\lambda_{43}y\right)+C_{44}\exp\left(\lambda_{44}y\right)+A_{4}sinh\left(My\right),$$

The coefficients in Equations (36)–(39) are given in detail in Appendix A, based on which analytical solutions for velocity in the PE-grafted microchannel can be derived.

For the electrolyte layer: 0≤y≤1−d, the axial velocity ${\tilde{u}}_{1}\left(x,y\right)$ and the vertical velocity ${\tilde{v}}_{1}\left(x,y\right)$ can be set as:

(40) $${\tilde{u}}_{1}\left(x,y\right)=\frac{\partial \phi_{1}\left(x,y\right)}{\partial y}=\frac{\partial g_{1}\left(y\right)}{\partial y}+\frac{\partial g_{2}\left(y\right)}{\partial y}cos\left(mx\right)$$

(41) $${\tilde{v}}_{1}\left(x,y\right)=-\frac{\partial \phi_{1}\left(x,y\right)}{\partial x}=-mg_{2}\left(y\right)sin\left(mx\right)$$

For the PEL: 1−d≤y≤1, the axial velocity ${\tilde{u}}_{2}\left(x,y\right)$ and the vertical velocity ${\tilde{v}}_{2}\left(x,y\right)$ can be set as:

(42) $${\tilde{u}}_{2}\left(x,y\right)=\frac{\partial \phi_{2}\left(x,y\right)}{\partial y}=\frac{\partial g_{3}\left(y\right)}{\partial y}+\frac{\partial g_{4}\left(y\right)}{\partial y}cos\left(mx\right)$$

(43) $${\tilde{v}}_{2}\left(x,y\right)=-\frac{\partial \phi_{2}\left(x,y\right)}{\partial x}=-mg_{4}\left(y\right)sin\left(mx\right)$$

### 2.3. Concentration Distribution

The transport of solute in the microchannel is governed by the two-dimensional unsteady convection–diffusion equation. Considering the two-dimensional velocity field obtained in Section 2.2, the dimensional governing equation is expressed as

(44) $$\frac{\partial C^{*}}{\partial t^{*}}+u^{*}\frac{\partial C^{*}}{\partial x^{*}}+v^{*}\frac{\partial C^{*}}{\partial y^{*}}=D\left(\frac{\partial^{2}C^{*}}{\partial {x^{*}}^{2}}+\frac{\partial^{2}C^{*}}{\partial {y^{*}}^{2}}\right),$$

where $C^{*}$ is the solute concentration and *D* is the diffusion coefficient.

The initial and boundary conditions are as follows:

(45) $$\left\{\begin{array}{c} \;C^{*}\left(0,y^{*},t^{*}\right)=C_{0},\\ \;C^{*}\left(x^{*},y^{*},0\right)=0,\;\left(x^{*}>0\right),\\ \;{\left.\frac{\partial C^{*}}{\partial y^{*}}\right|}_{y^{*}=\pm H}=0 \end{array}\right.$$

Equation (45) describes that the concentration at the inlet is constant, the initial concentration is zero, and the walls are impermeable.

By using $L/u_{ref}$ and $C_{0}$ as the time and concentration scales ($t=t^{*}/t_{0},$ $t_{0}=L/u_{ref},\;C=C^{*}/C_{0}$), the unsteady convection–diffusion equation is nondimensionalized, and the dimensionless concentration C(x,y,t) satisfies:

(46) $$\frac{\partial C}{\partial t}+u\frac{\partial C}{\partial x}+v\frac{\partial C}{\partial y}=\frac{1}{Pe}\left(\beta \frac{\partial^{2}C}{\partial x^{2}}+\frac{1}{\beta }\frac{\partial^{2}C}{\partial y^{2}}\right).$$

where $Pe=\frac{Hu_{ref}}{D}$ represents the Peclet number, representing the ratio of convective to diffusive transport.

The corresponding dimensionless boundary conditions are

(47) $$\left\{\begin{array}{c} \;C(0,y,t)=1,\\ \;C(x,y,0)=0,\;(x>0),\\ \;{\left.\frac{\partial C}{\partial y}\right|}_{y=\pm 1}=0. \end{array}\right.$$

The analytical solution of the governing convection–diffusion equation is difficult to obtain due to the strong coupling between convection and diffusion and the complex boundary conditions. The detailed discretization procedure and computational algorithm are provided in Appendix B.

## 3. Results and Discussions

The EOF and diffusion behavior of Maxwell fluid solutes in a microchannel with modulated charge surfaces under the influence of an electric field are studied. Some dimensionless parameters play significant roles in the fluid motion in the microchannel, such as the modulation parameter α, the Deborah number De, the oscillating Reynolds number $Re_{\omega }$, and so on. According to previous research, the physical parameters are as follows: H∼ 10–100 μm, ρ∼ $10^{3}$ kg $m^{-3}$, η∼ $10^{-3}$ Pa s. The parametric regions of the frequency of the external electric field change from 0 to 1.6 kHz, corresponding to the oscillation frequency ω from 0 to $10^{4}$ $s^{-1}$. The oscillating Reynolds number $Re_{\omega }$ can be evaluated by changing from 0 to 100. The molecular diffusion coefficient *D* varies from $O(10^{-8})$ $m^{2}$ $s^{-1}$ to $O(10^{-9})$ $m^{2}$ $s^{-1}$. The relaxation time should be shorter than the oscillation period, so λ<2π/ω or De<2π. The Peclet number Pe varies from 0.1 to 1. The initial concentration $C_{0}$ = 10^3^ mol · $m^{-3}$ [33].

### 3.1. Electrical Potential Field

**Figure 2:**
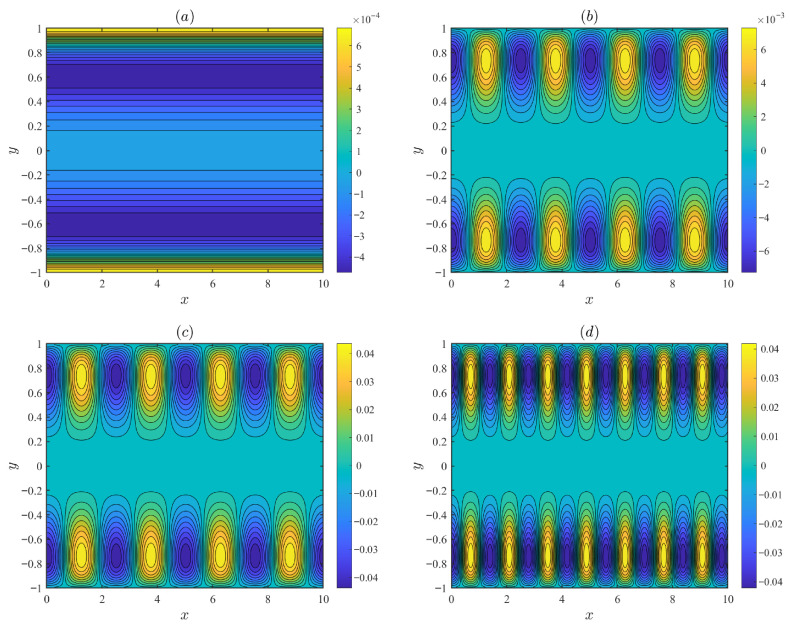
The variations in the streamlines for different values of the modulated parameter α, the mode frequency *m*, and the modulation potential amplitude $\xi_{0}$: (**a**) α = 0, $\xi_{0}$ = 0.1. (**b**) α = 1.5, *m* = 2.5, $\xi_{0}$ = 0.1. (**c**) α = 1.5, *m* = 2.5, $\xi_{0}$ = 0.6. (**d**) α = 1.5, *m* = 4.5, $\xi_{0}$ = 0.6.

Figure 2 illustrates the distributions of the streamlines under varying parameters, including the modulated parameter α, the mode frequency *m*, and the modulation potential amplitude $\xi_{0}$. The fixed parameters are set as follows: *d* = 0.1, λ=0.2, $\lambda_{F}$ = 0.5, $\alpha_{0}$ = 0.15, β = 0.1, De = 3, and $Re_{\omega }$ = 5. In Figure 2a, when α = 0, the wall potential is uniform and the fluid flows purely along the x-axis without vertical velocity component. In Figure 2b–d, when α≠0, the recirculating flow is intensified. Charge modulation induces a vertical velocity that leads to the formation of recirculating flow. As the mode frequency *m* increases, the vortical period decreases, and the flow intensified.

**Figure 3:**
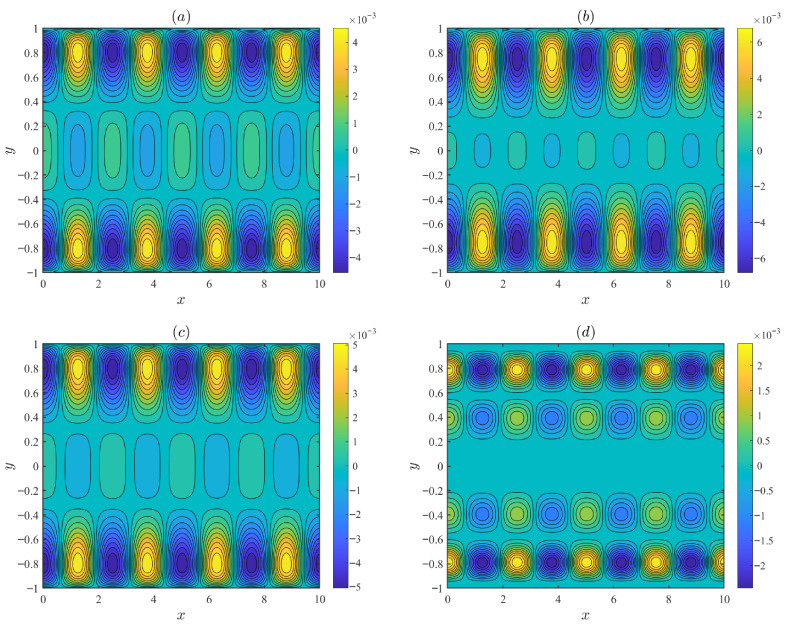
The variations in the streamlines for different values of the oscillating Reynolds number $Re_{\omega }$ and the Deborah number De: (**a**) $Re_{\omega }$ = 5, De = 0.3. (**b**) $Re_{\omega }$ = 5, De = 3. (**c**) $Re_{\omega }$ = 15, De = 0.3. (**d**) $Re_{\omega }$ = 50, De = 5.

Figure 3 depicts the streamline distributions under different values of the oscillating Reynolds number $Re_{\omega }$ and the Deborah number De, with fixed parameters *d* = 0.1, λ=0.2, $\lambda_{F}$ = 0.5, $\alpha_{0}$ = 0.15, *m* = 2.5, $\xi_{0}$ = 0.1, α = 1.5 and β = 0.1. As the Deborah number De increases, the vortices in the circular flow gradually become stronger. As the oscillatory Reynolds number $Re_{\omega }$ increases, the strength of the recirculating flow grows. However, when the oscillating Reynolds number $Re_{\omega }$ continues to increase, the number of vortices rises while their intensity decreases. This non-monotonic trend occurs because, at higher oscillating Reynolds number $Re_{\omega }$, the oscillation frequency becomes sufficiently high that the fluid cannot complete its hydrodynamic response within one oscillation period.

**Figure 4:**
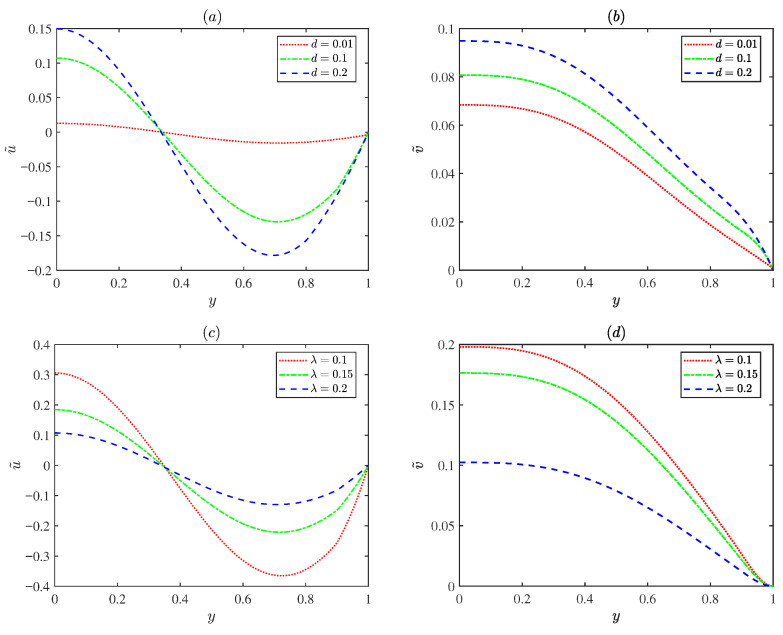
The variations in the dimensionless velocity amplitude for different values of PEL thickness *d* and EDL thickness λ with $\lambda_{F}$ = 0.5, $\alpha_{0}$ = 0.25, *m* = 5, $\xi_{0}$ = 0.25, α = 1.5, β = 0.1, $Re_{\omega }$ = 5 and De = 3 at *x* = π/2: (**a**,**b**) λ = 0.2. (**c**,**d**) *d* = 0.1.

Figure 4 displays variations in the normalized EOF axial velocity amplitude $\tilde{u}$ and the transverse velocity amplitude $\tilde{v}$ with the PEL thickness *d* (*d* = 0.01, 0.1, 0.2) and the EDL thickness λ (λ = 0.1, 0.15, 0.2). As shown in Figure 4a,b, the velocity increases as the PEL thickness *d* increases. Physically, the PEL layer carries fixed charges; increasing the PEL thickness results in an enhanced electrostatic potential. Under the applied electric field, the higher potential exerts a stronger electroosmotic body force on the EDL, thereby accelerating the fluid flow. Figure 4c,d show the velocity decreases as the EDL thickness λ increases. An increase in λ implies a rise in ion density within the EDL, indicating ion accumulation, which leads to high resistance to flow and a reduction in the velocity.

**Figure 5:**
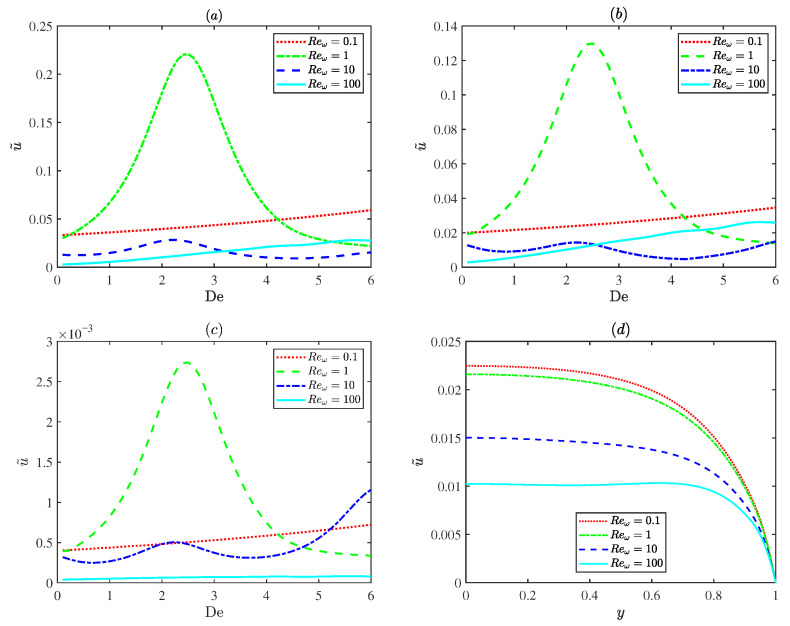
The variations in the dimensionless axial velocity amplitude for different values of oscillating Reynolds number $Re_{\omega }$ (0.1, 1, 10 and 100) with λ = 0.2, $\lambda_{F}$ = 0.5, $\alpha_{0}$ = 0.25, *m* = 5, $\xi_{0}$ = 0.25, β = 0.1 at *x* = π/2: (**a**) α=1.5, d=0.1 at y=0. (**b**) α=0.01, d=0.1 at y=0. (**c**) α=1.5, d=0.01 at y=0. (**d**) α = 1.5, *d* = 0.1, De = 0.01.

Figure 5 describes variations in the dimensionless EOF velocity amplitude for different oscillating Reynolds numbers ($Re_{\omega }=0.1,1,10,100$). In Figure 5a–c, at an oscillatory Reynolds number of 1 ($Re_{\omega }=1$), the plot exhibits a distinct resonance behavior, where the velocity reaches a distinct peak due to resonance. Although weak surface charge modulation (α=0.01 in Figure 5b) and the thin PEL limit (d = 0.01 in Figure 5c) both reduce the velocity magnitude, the resonance behavior still persists. However, for the small Deborah number De (De=0.01 in Figure 5d), the Maxwell fluid behaves like a Newtonian fluid and the resonance disappears. This demonstrates that the resonance is an intrinsic characteristic of Maxwell fluids. Physically, the oscillatory Reynolds number is associated with the frequency of the applied electric field. And the Deborah number reflects the intrinsic relaxation property of the fluid. Resonance occurs when the frequency of the applied oscillatory electric field becomes close to the intrinsic relaxation frequency of the Maxwell fluid, resulting in a significant enhancement of the EOF velocity.

**Figure 6:**
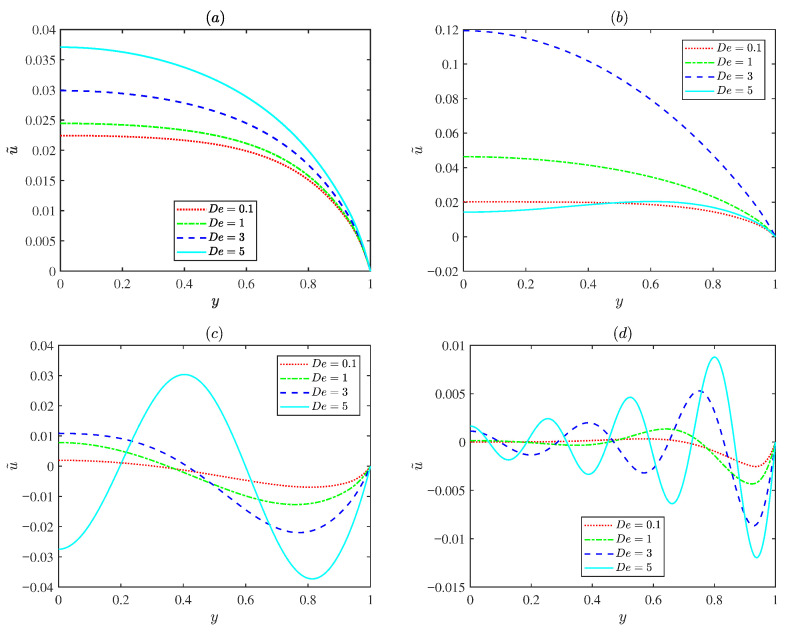
The variations in the dimensionless axial velocity amplitude for different values of the Deborah number De (0.1, 1, 3 and 5) with *d* = 0.1, λ=0.2, $\lambda_{F}$ = 0.5, $\alpha_{0}$ = 0.15, *m* = 2.5, $\xi_{0}$ = 0.1, α = 1.5, β = 0.1 at *x* = π/2: (**a**) $Re_{\omega }$ = 0.1. (**b**) $Re_{\omega }$ = 1. (**c**) $Re_{\omega }$ = 10. (**d**) $Re_{\omega }$ = 100.

Figure 6 indicates the variations in the normalized EOF velocity amplitude $\tilde{u}$ for various Deborah numbers (De = 0.1, 1, 3, and 5) under different oscillating Reynolds numbers $Re_{\omega }$. For low oscillating Reynolds number $Re_{\omega }$, the flow exhibits a classical plug-like Helmholtz Smoluchowski profile, and the velocity varies with the Deborah number De. For the prescribed Deborah number De, an increase in the oscillating Reynolds number $Re_{\omega }$ leads to rapid oscillating EOF velocity profiles and the EOF velocity amplitude decreases. This is because the diffusion time scale is much longer than the oscillation time period and the fluid does not have sufficient time to diffuse. Figure 6b presents the velocity remaining nearly uniform due to viscous dominance at small Deborah numbers. As the Deborah number increases to about three (De = 3), the velocity near the channel center increases significantly, indicating an elastic resonance. When the Deborah number De further increases, the amplitude decreases again because excessive elasticity delays the fluid response. This spatial behavior is consistent with the resonance peak shown in Figure 5a.

**Figure 7:**
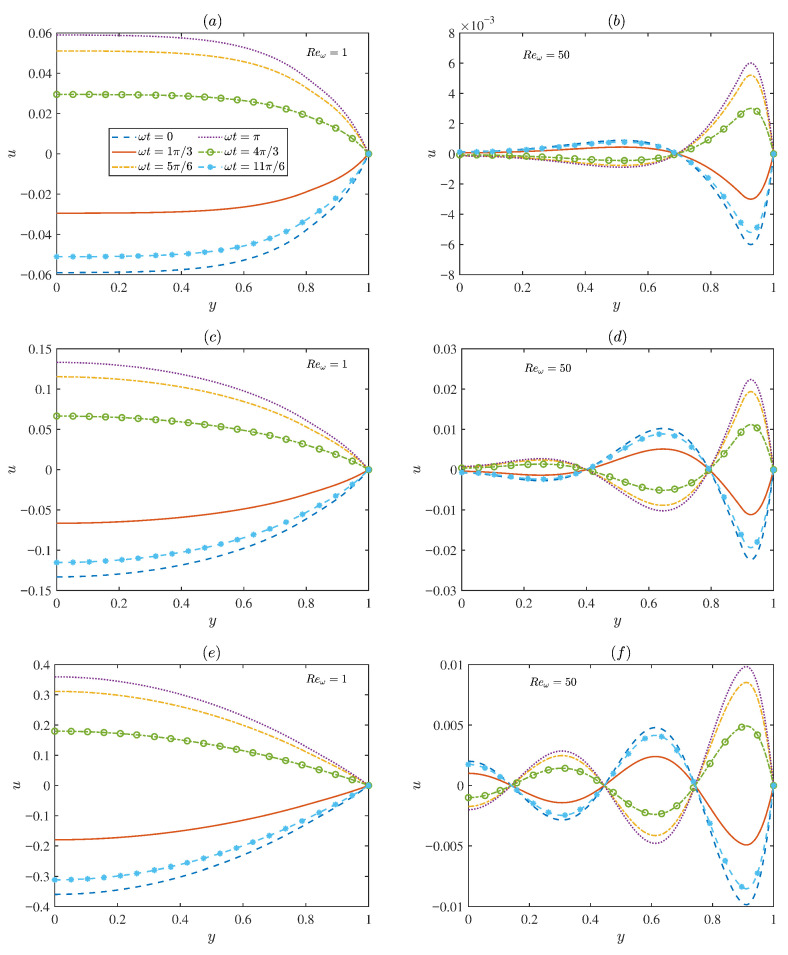
The variations in the dimensionless axial velocity for different values of ωt (ωt = 0, π/3, 5π/6, π, 4π/3, 11π/6) under different oscillating Reynolds numbers $Re_{\omega }$ ($Re_{\omega }$ = 1, 50) and the Deborah numbers De, with *d* = 0.1, λ = 0.2, $\lambda_{F}$ = 0.5, $\alpha_{0}$ = 0.15, *m* = 2.5, $\xi_{0}$ = 0.15, α = 1.5, β = 0.1 at *x* = π/2: (**a**,**b**) De = 0.1. (**c**,**d**) De = 1. (**e**,**f**) De = 2.

Figure 7 presents the temporal evolution of the dimensionless velocity for two representative oscillating Reynolds numbers $Re_{\omega }$ ($Re_{\omega }$ = 1 and 50). At low oscillating Reynolds number $Re_{\omega }$ (Figure 7a,c,e), the flow is predominantly governed by viscous effects, leading to smooth distributions with relatively large amplitudes. In contrast, for higher oscillating Reynolds number $Re_{\omega }$ (Figure 7b,d,f), the velocity distribution gradually exhibits oscillatory characteristics. The flow field is restricted to a narrow region near the microchannel walls, where distinct velocity oscillations occur. In the region far from the wall, the velocity oscillation amplitude approaches zero. This is because, at high electric field frequencies, the diffusion time is longer than the oscillation period. Overall, Figure 7 demonstrates the strong coupling between viscous diffusion and elastic relaxation in determining the EOF structure.

### 3.2. Concentration Field

**Figure 8:**
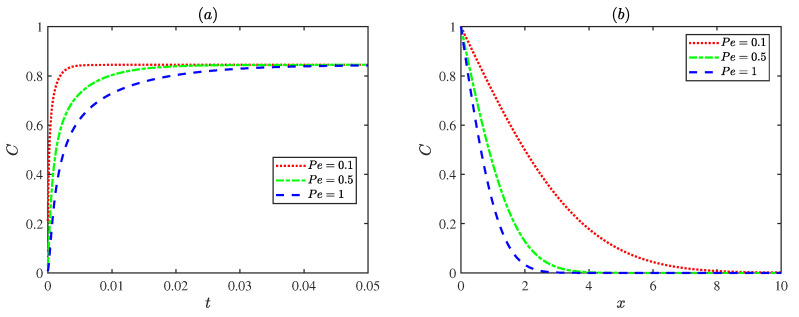
The dimensionless concentration distributions for different Peclet numbers Pe with ωt = π, De = 3, $Re_{\omega }$ = 1: (**a**) x=π/4, *y* = 0. (**b**) *y* = 0, *t* = 0.6.

Figure 8 illustrates the variations in concentrations with the Peclet number Pe. As the Peclet number Pe increases, the solute diffusion transport within the microchannel becomes progressively weaker. Physically, a low Peclet number Pe indicates that the diffusion term dominates the flow, leading to a more uniform concentration distribution. In contrast, at higher Peclet numbers Pe, convective transport becomes dominant, resulting in a steeper concentration gradient and lower concentration levels within the channel.

**Figure 9:**
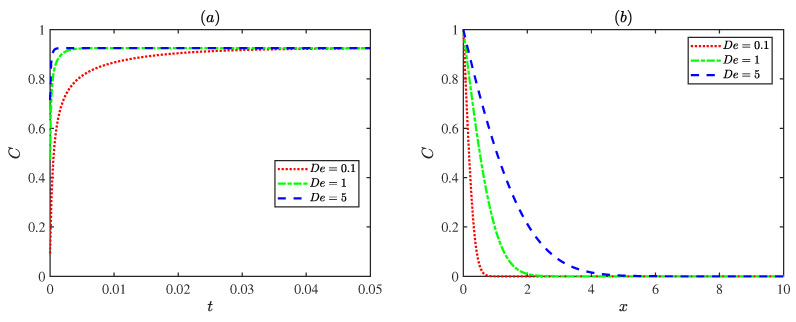
The dimensionless concentration distributions for different Deborah numbers De with ωt = π, Pe = 1, $Re_{\omega }$ = 1 at (**a**) x=π/4, *y* = 0. (**b**) *y* = 0, *t* = 0.6.

Figure 9 shows fluctuations in concentrations with the Deborah number De. When the Deborah number De is small, the fluid tends to behave like a Newtonian fluid, the elastic effect is weak, and solute diffusion transport is mainly governed by diffusion. As the Deborah number De increases, the fluid elasticity becomes stronger and the fluid responds more rapidly to the external field, accelerating the concentration transport process and reaching the steady state earlier. This indicates that the elastic effect of the fluid can enhance the rate of solute diffusion transport.

**Figure 10:**
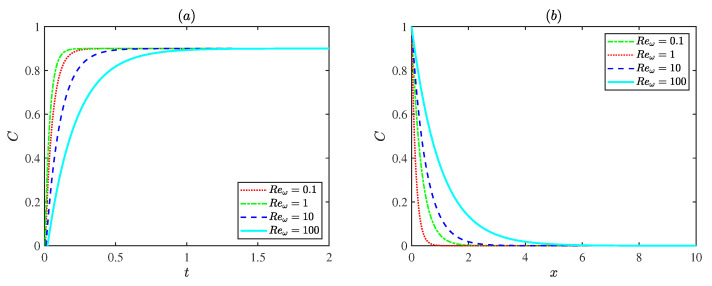
The dimensionless concentration distributions for different Reynolds numbers $Re_{\omega }$ with ωt = π, Pe = 1, De = 3 at (**a**) x=π/4, *y* = 0. (**b**) *y* = 0, *t* = 0.6.

Figure 10 presents the influence of the oscillating Reynolds number $Re_{\omega }$ on the dimensionless concentration distribution at a fixed Deborah number De (De = 3). For De=3, the case with $Re_{\omega }$ = 1 reaches steady state most rapidly. This is attributed to the higher flow velocity at this condition, which enhances convective transport and accelerates solute mixing. The concentration variation slows down at both smaller and larger oscillating Reynolds numbers $Re_{\omega }$. Weak convection limits transport at low oscillating Reynolds number $Re_{\omega }$, while strong inertial oscillations restrict solute accumulation at high oscillating Reynolds number $Re_{\omega }$. In general, increasing the oscillating Reynolds number $Re_{\omega }$ results in a more gradual spatial decay of concentration, indicating that stronger oscillations promote solute dispersion and produce a more uniform concentration field along the channel.

## 4. Conclusions

This study investigated the EOF and solute diffusion transport of the Maxwell fluid under an oscillatory electric field and modulated charged surfaces. The results show that the modulation of the wall potential and the oscillation frequency jointly control the flow pattern and the associated dynamic behavior. Electrokinetic parameters play a crucial role: a thicker PEL enhances the driving force, while a thicker EDL increases resistance. The elastic effect, represented by the Deborah number De, accelerates the transport of solute by improving the dynamic response of the fluid. Resonance appears when the oscillating Reynolds number $Re_{\omega }$ ($Re_{\omega }=1$), leading to a higher amplitude of the velocity. The results confirm that the resonance is fundamentally governed by the viscoelasticity of Maxwell fluid, rather than electrokinetic or interfacial effects. When the oscillating Reynolds number $Re_{\omega }$ takes other values, viscous damping weakens the oscillatory flow. Collectively, these findings illuminate the interplay between elastic resonance and oscillatory electroosmotic transport, providing physical insights for the optimization of microfluidic systems involving viscoelastic and electrokinetic effects.

## Data Availability

The original contributions presented in this study are included in the article. Further inquiries can be directed to the corresponding author.

## Glossary

**Symbols**	**Description**	**Symbols**	**Description**
$C^{*}$	Concentration	$u_{ref}$	Reference velocity scale
$C_{0}$	Initial concentration	$v^{*}$	Velocity in *y*-direction
*C*	Dimensionless concentration	*v*	Dimensionless velocity in *y*-direction
*D*	Molecular diffusion coefficient	*Z*	Valence of fixed-charge ions in PEL
De	Deborah number	*z*	Absolute value of ionic valence
$d^{*}$	Thickness of PEL	α	Modulation parameter
*d*	Dimensionless thickness of PEL	$\alpha_{0}$	Dimensionless resistance parameter within the PEL
*e*	Elementary charge	β	Dimensionless ratio of the half-height to the length of the microchannel
$E_{x^{*}}$	Electric field	ϵ	Electric permittivity of electrolyte solution
$E_{0}$	Amplitude of electric field	η	Zero shear viscosity
*H*	Half height of the channel	$\mu_{c}$	Drag coefficient in PEL
$K_{B}$	Boltzmann constant	$\psi^{*}$	Electric potential
*L*	Length of channel	$\psi_{s}$	Reference electric potential
$m^{*}$	Mode frequency	ψ	Dimensionless electric potential
*m*	Dimensionless mode frequency	ρ	Density of fluid
*N*	Number concentration of fixed-charge ions in PEL	$\rho_{e}$	Net charge density of electrolyte
$n_{0}$	Bulk ion concentration	$\xi_{0}^{*}$	Modulated potential amplitude
$P^{*}$	Pressure	$\xi_{0}$	Dimensionless modulated potential amplitude
*P*	Dimensionless pressure	λ	Dimensionless thickness of EDL
Pe	Peclet number	$\lambda^{*}$	Thickness of EDL
$Re_{\omega }$	Oscillating Reynolds number	$\lambda_{F}$	Dimensionless equivalent EDL thickness in PEL
$t_{m}$	Relaxation time scale	$\lambda_{F}^{*}$	Equivalent EDL thickness in PEL
$t^{*}$	Time	ω	Oscillation frequency of the applied electric field
*t*	Dimensionless time	$\tau_{yx}^{*}$	Shear stress in yx-plane
$u^{*}$	Velocity in *x*-direction	τ	Deviatoric stress tensor
*u*	Dimensionless velocity in *x*-direction		

## Appendix A. Mathematical Formulas for Equations (36)–(39)

The characteristic roots in Equations (36)–(39) are the respective solutions to the following equations:

(A1) $$\lambda_{1j}^{4}-iRe_{\omega }\varphi \lambda^{2}=0,\;j=1,2,3,4,$$

(A2) $$\lambda_{2j}^{4}-\left(2m^{2}\beta^{2}+iRe_{\omega }\varphi \right)\lambda^{2}+\left(m^{4}\beta^{4}+im^{2}\beta^{2}Re_{\omega }\varphi \right)=0,\;j=1,2,3,4,$$

(A3) $$\lambda_{3j}^{4}-\left(iRe_{\omega }\varphi +\alpha_{0}^{2}\varphi \right)\lambda^{2}=0,\;j=1,2,3,4,$$

(A4) $$\lambda_{4j}^{4}-\left(2m^{2}\beta^{2}+iRe_{\omega }\varphi +\alpha_{0}^{2}\varphi \right)\lambda^{2}+\left(m^{4}\beta^{4}+im^{2}\beta^{2}Re_{\omega }\varphi +\alpha_{0}^{2}m^{2}\beta^{2}\varphi \right)=0,\;j=1,2,3,4,$$

Among them $\lambda_{11}=\lambda_{12}=0$, $\lambda_{13}=-\lambda_{14}$, $\lambda_{21}=-\lambda_{22}$, $\lambda_{23}=-\lambda_{24}$, $\lambda_{31}=\lambda_{32}=0$, $\lambda_{33}=-\lambda_{34}$, $\lambda_{41}=-\lambda_{42}$, $\lambda_{43}=-\lambda_{44}$. The coefficients in Equations (36)–(39) are expressed as follows:

(A5) $$A_{1}=\frac{\varphi \lambda }{1-iRe_{\omega }\varphi \lambda^{2}}\left[\frac{-\sinh(D)\sinh(1/\lambda )-1}{\Lambda^{2}\cosh\left(1/\lambda \right)}+\frac{\cosh(D)}{\Lambda^{2}}\right],$$

(A6) $$A_{2}=\frac{\varphi \lambda^{2}}{1-iRe_{\omega }\varphi \lambda^{2}}\frac{M\alpha \xi_{0}}{\cosh(M)},$$

(A7) $$A_{31}=\frac{\varphi \lambda }{1-\left(iRe_{\omega }\varphi +\alpha_{0}^{2}\varphi \right)\lambda^{2}}\left[\frac{\Lambda^{2}\xi_{0}-\sinh\left(D\right)\sinh\left(1/\lambda \right)}{\Lambda^{2}\cosh\left(1/\lambda \right)}\right],$$

(A8) $$A_{32}=\frac{\varphi \lambda }{1-\left(iRe_{\omega }\varphi +\alpha_{0}^{2}\varphi \right)\lambda^{2}}\left[\frac{\sinh(D)}{\Lambda^{2}}\right],$$

(A9) $$A_{4}=\frac{\varphi \lambda^{2}}{1-\left(iRe_{\omega }\varphi +\alpha_{0}^{2}\varphi \right)\lambda^{2}}\frac{M\alpha \xi_{0}}{\cosh(M)},$$

In addition, the coefficients of the exponential term satisfy the following algebraic equations: $C_{11}=C_{12}=0$ and $C_{31}=C_{32}=0$. The other coefficients are derived from the equation:

(A10) $$\hat{\Gamma_{l}}\hat{C_{l}}=\hat{X_{l}},\;l=1,2.$$

Solving Equation (A10) leads to the coefficients $\hat{C_{l}},\;l=1,2$, the analytical solution to the stream function ϕ(x,y) and velocity is obtained.

The specific array of solutions to the above equations is given below:

(A11) $$\hat{C_{1}}={\left(C_{13},C_{14},C_{33},C_{34}\right)}^{T},$$

(A12) $$\hat{C_{2}}={\left(C_{21},C_{22},C_{23},C_{24},C_{41},C_{42},C_{43},C_{44}\right)}^{T},$$

(A13) $$\hat{\Gamma_{1}}=\left(\begin{array}{cccc} \lambda_{11}^{2} & \lambda_{12}^{2} & 0 & 0\\ 0 & 0 & \lambda_{31}\exp\left(\lambda_{31}\right) & \lambda_{32}\exp\left(\lambda_{32}\right)\\ \lambda_{11}\exp\left[\lambda_{11}\left(1-d\right)\right] & \lambda_{12}\exp\left[\lambda_{12}\left(1-d\right)\right] & -\lambda_{31}\exp\left[\lambda_{31}\left(1-d\right)\right] & -\lambda_{31}\exp\left[\lambda_{32}\left(1-d\right)\right]\\ \lambda_{11}^{2}\exp\left[\lambda_{11}\left(1-d\right)\right] & \lambda_{12}^{2}\exp\left[\lambda_{11}\left(1-d\right)\right] & -\lambda_{31}^{2}\exp\left[\lambda_{31}\left(1-d\right)\right] & -\lambda_{32}^{2}\exp\left[\lambda_{32}\left(1-d\right)\right] \end{array}\right),$$

(A14) $$\hat{X_{1}}=\left(\begin{array}{c} 0\\ -\frac{A_{31}\cosh\left(1/\lambda \right)+A_{32}\sinh\left(1/\lambda \right)}{\lambda }\\ \frac{\left(-A_{1}+A_{31}\right)\cosh\left(D\right)+A_{32}\sinh\left(D\right)}{\lambda }\\ \frac{\left(-A_{1}+A_{31}\right)\sinh\left(D\right)+A_{32}\cosh\left(D\right)}{\lambda^{2}} \end{array}\right).$$

(A15) $$\hat{\Gamma_{2}}=\left(\begin{array}{cc} \hat{\Gamma_{21}} & 0\\ 0 & \hat{\Gamma_{22}}\\ \hat{\Gamma_{23}} & \hat{\Gamma_{24}} \end{array}\right),$$

(A16) $$\hat{\Gamma_{21}}=\left(\begin{array}{cccc} \lambda_{21} & \lambda_{22} & \lambda_{23} & \lambda_{24}\\ \lambda_{21} & -2\lambda_{22} & 3\lambda_{23} & -4\lambda_{24}\\ \lambda_{21}^{2} & \lambda_{22}^{2} & \lambda_{23}^{2} & \lambda_{24}^{2} \end{array}\right),$$

(A17) $$\hat{\Gamma_{22}}=\left(\begin{array}{cccc} \exp(\lambda_{41}) & \exp(\lambda_{42}) & \exp(\lambda_{43}) & \exp(\lambda_{44})\\ \lambda_{41}\exp\left(\lambda_{41}\right) & \lambda_{42}\exp\left(\lambda_{42}\right) & \lambda_{43}\exp\left(\lambda_{43}\right) & \lambda_{44}\exp\left(\lambda_{44}\right) \end{array}\right),$$

(A18) $$\hat{\Gamma_{23}}=\left(\begin{array}{cccc} \exp[\lambda_{21}\left(1-d\right)] & \exp[\lambda_{22}\left(1-d\right)] & \exp[\lambda_{23}\left(1-d\right)] & \exp[\lambda_{24}\left(1-d\right)]\\ \lambda_{21}\exp\left[\lambda_{21}\left(1-d\right)\right] & \lambda_{22}\exp\left[\lambda_{22}\left(1-d\right)\right] & \lambda_{23}\exp\left[\lambda_{23}\left(1-d\right)\right] & \lambda_{24}\exp\left[\lambda_{24}\left(1-d\right)\right]\\ \lambda_{21}^{2}\exp\left[\lambda_{21}\left(1-d\right)\right] & \lambda_{22}^{2}\exp\left[\lambda_{22}\left(1-d\right)\right] & \lambda_{23}^{2}\exp\left[\lambda_{23}\left(1-d\right)\right] & \lambda_{24}^{2}\exp\left[\lambda_{24}\left(1-d\right)\right] \end{array}\right),$$

(A19) $$\hat{\Gamma_{24}}=\left(\begin{array}{cccc} -\exp[\lambda_{41}\left(1-d\right)] & -\exp[\lambda_{42}\left(1-d\right)] & -\exp[\lambda_{43}\left(1-d\right)] & -\exp[\lambda_{44}\left(1-d\right)]\\ -\lambda_{41}\exp\left[\lambda_{41}\left(1-d\right)\right] & -\lambda_{42}\exp\left[\lambda_{42}\left(1-d\right)\right] & -\lambda_{43}\exp\left[\lambda_{43}\left(1-d\right)\right] & -\lambda_{44}\exp\left[\lambda_{44}\left(1-d\right)\right]\\ -\lambda_{41}^{2}\exp\left[\lambda_{41}\left(1-d\right)\right] & -\lambda_{42}^{2}\exp\left[\lambda_{42}\left(1-d\right)\right] & -\lambda_{43}^{2}\exp\left[\lambda_{43}\left(1-d\right)\right] & -\lambda_{44}^{2}\exp\left[\lambda_{44}\left(1-d\right)\right] \end{array}\right),$$

(A20) $$\hat{X_{2}}=\left(\begin{array}{c} -MA_{2}\\ MA_{2}\\ 0\\ -A_{4}\sinh\left(M\right)\\ -MA_{4}\cosh\left(M\right)\\ \left(A_{4}-A_{2}\right)\sinh\left[M\left(1-d\right)\right]\\ M\left(A_{4}-A_{2}\right)\cosh\left[M\left(1-d\right)\right]\\ M^{2}\left(A_{4}-A_{2}\right)\sinh\left[M\left(1-d\right)\right] \end{array}\right).$$

## Appendix B. Numerical Algorithm for Equations (46) and (47)

The convection–diffusion Equation (46) and boundary conditions (47) are solved numerically using a semi-implicit finite difference method on a uniform grid. The computational domain is discretized with grid spacings and time steps given by Δx=0.1, Δy=0.1, $\Delta t=10^{-4}$.

In the present scheme, the diffusion terms are treated implicitly, while the convection terms are discretized explicitly. As a result, numerical stability is governed by the explicit convection terms. The time step is chosen to satisfy the Courant–Friedrichs–Lewy (CFL) condition [53]:

(A21) $$\frac{|u|\Delta t}{\Delta x}+\frac{|v|\Delta t}{\Delta y}\leq 1.$$

The spatial derivatives are approximated using second-order central differences, and the time derivative is discretized using a first-order forward difference. Specifically,

(A22) $$\frac{\partial C}{\partial t}\approx \frac{C_{i,j}^{k+1}-C_{i,j}^{k}}{\Delta t},\;\frac{\partial C}{\partial x}\approx \frac{C_{i+1,j}^{k}-C_{i-1,j}^{k}}{2\Delta x},\;\frac{\partial C}{\partial y}\approx \frac{C_{i,j+1}^{k}-C_{i,j-1}^{k}}{2\Delta y},$$

(A23) $$\frac{\partial^{2}C}{\partial x^{2}}\approx \frac{C_{i+1,j}^{k+1}-2C_{i,j}^{k+1}+C_{i-1,j}^{k+1}}{\Delta x^{2}},\;\frac{\partial^{2}C}{\partial y^{2}}\approx \frac{C_{i,j+1}^{k+1}-2C_{i,j}^{k+1}+C_{i,j-1}^{k+1}}{\Delta y^{2}}.$$

Substituting the above discretizations into the governing equation yields the semi-implicit difference scheme:

(A24) $$\begin{array}{cc} C_{i,j}^{k+1}=C_{i,j}^{k} & -\Delta t\left[u_{i,j}^{k}\frac{C_{i+1,j}^{k}-C_{i-1,j}^{k}}{2\Delta x}+v_{i,j}^{k}\frac{C_{i,j+1}^{k}-C_{i,j-1}^{k}}{2\Delta y}\right]\\ \; & +\Delta t\frac{1}{Pe}\left[\beta \frac{C_{i+1,j}^{k+1}-2C_{i,j}^{k+1}+C_{i-1,j}^{k+1}}{\Delta x^{2}}+\frac{1}{\beta }\frac{C_{i,j+1}^{k+1}-2C_{i,j}^{k+1}+C_{i,j-1}^{k+1}}{\Delta y^{2}}\right]. \end{array}$$

At each time step, Equation (A24) is rearranged to collect all unknown terms at time level k+1 on the left-hand side. This leads to a system of coupled algebraic equations in which each grid node is connected with its four nearest neighbors due to the implicit discretization of the diffusion terms. Therefore, the resulting discretization forms a sparse linear system for the unknown concentration field $C^{k+1}$.

The unknown concentration vector is ordered in a column-wise manner as

(A25) $$C^{k+1}={\left[C_{1,1}^{k+1},\ldots ,C_{N_{x},1}^{k+1},C_{1,2}^{k+1},\ldots ,C_{N_{x},N_{y}}^{k+1}\right]}^{T}.$$

The discrete equations over the entire computational domain can be expressed in matrix form as

(A26) $$AC^{k+1}=b^{k},$$

where A is the coefficient matrix determined by the implicit diffusion operator, and $b^{k}$ contains all explicitly evaluated convection terms as well as known values at time level *k*. The matrix A has a standard five-point stencil structure, leading to a sparse and diagonally dominant system.

For interior grid nodes, the coefficient matrix A has a block tridiagonal structure given by

(A27) $$A=\left[\begin{array}{ccccc} B & -r_{y}I & 0 & \ldots & 0\\ -r_{y}I & B & -r_{y}I & \ldots & 0\\ 0 & -r_{y}I & B & \ldots & 0\\ \vdots & \vdots & \vdots & \ddots & -r_{y}I\\ 0 & 0 & 0 & -r_{y}I & B \end{array}\right],$$

where I is the $N_{x}\times N_{x}$ identity matrix, and

(A28) $$B=\left[\begin{array}{ccccc} 1+2r_{x}+2r_{y} & -r_{x} & 0 & \ldots & 0\\ -r_{x} & 1+2r_{x}+2r_{y} & -r_{x} & \ldots & 0\\ 0 & -r_{x} & 1+2r_{x}+2r_{y} & \ldots & 0\\ \vdots & \vdots & \vdots & \ddots & -r_{x}\\ 0 & 0 & 0 & -r_{x} & 1+2r_{x}+2r_{y} \end{array}\right],$$

while the matrix rows corresponding to boundary nodes are modified according to the prescribed boundary conditions, where a Dirichlet condition C(0,y,t)=1 is imposed at the inlet, and zero-gradient (Neumann) boundary conditions are applied at the upper and lower channel walls.

The coefficient parameters are defined as

(A29) $$r_{x}=\frac{\beta \Delta t}{Pe\;\Delta x^{2}},\;r_{y}=\frac{\Delta t}{Pe\;\beta \;\Delta y^{2}}.$$

The $b^{k}$ is given by

(A30) $$b^{k}={\left[b_{1,1}^{k},\ldots ,b_{N_{x},1}^{k},\ldots b_{N_{x},N_{y}}^{k}\right]}^{T},$$

where each element is

(A31) $$b_{i,j}^{k}=C_{i,j}^{k}-\Delta t\left[u_{i,j}^{k}\frac{C_{i+1,j}^{k}-C_{i-1,j}^{k}}{2\Delta x}+v_{i,j}^{k}\frac{C_{i,j+1}^{k}-C_{i,j-1}^{k}}{2\Delta y}\right].$$

## References

[1] Gardeniers H., Van Den Berg A. (2004). Micro- and nanofluidic devices for environmental and biomedical applications. Int. J. Environ. Anal. Chem..

[2] Bow H., Fu J., Han J. (2008). Decreasing effective nanofluidic filter size by modulating electrical double layers: Separation enhancement in microfabricated nanofluidic filters. Electrophoresis.

[3] Chen X., Zhang S., Zhang L., Yao Z., Chen X., Zheng Y., Liu Y. (2017). Applications and theory of electrokinetic enrichment in micro-nanofluidic chips. Biomed. Microdevices.

[4] Song Y., Zhang J., Li D. (2017). Microfluidic and Nanofluidic Resistive Pulse Sensing: A Review. Micromachines.

[5] Oćwieja M., Barbasz A., Wasilewska M., Smoleń P., Duraczyńska D., Napruszewska B.D., Kozak M., Węgrzynowicz A. (2024). Surface Charge-Modulated Toxicity of Cysteine-Stabilized Silver Nanoparticles. Molecules.

[6] Li Y., Chen Z., Huhe C., Su Y., Xing H. (2024). Numerical Investigation of Flow Boiling in Interconnected Microchannels at Varying Mass Fluxes. Energies.

[7] Jang H.S., Park D.S. (2010). Microfabrication of Microchannels for Fuel Cell Plates. Sensors.

[8] Chen W.L. (2021). Electroosmosis and electric conduction of electrolyte solutions in charge-regulating fibrous media. Colloids Interfaces.

[9] Abdelmalek Z., D’Orazio A., Karimipour A. (2020). The effect of nanoparticle shape and microchannel geometry on fluid flow and heat transfer in a porous microchannel. Symmetry.

[10] Xia G.D., Jiang J., Wang J., Zhai Y.L., Ma D.D. (2015). Effects of different geometric structures on fluid flow and heat transfer performance in microchannel heat sinks. Int. J. Heat Mass Transf..

[11] Yu H., Li T., Zeng X., He T., Mao N. (2022). A critical review on geometric improvements for heat transfer augmentation of microchannels. Energies.

[12] Kose H.A., Yildizeli A., Cadirci S. (2022). Parametric study and optimization of microchannel heat sinks with various shapes. Appl. Therm. Eng..

[13] Banerjee D., Pati S., Biswas P. (2023). Analytical study of pulsatile mixed electroosmotic and shear-driven flow in a microchannel with a slip-dependent zeta potential. Appl. Math. Mech.-Engl. Ed..

[14] Zhao Q., Xu H., Tao L. (2021). Two-layer nanofluid flow and heat transfer in a horizontal microchannel with electric double layer effects and magnetic field. Int. J. Numer. Methods Heat Fluid Flow.

[15] De Moor T., Lagae L., Van Hoof C., Liu C., Van Roy W. (2023). Electric field gradient focusing with electro-osmotic flow to reduce analyte dispersion: Concept and numerical investigation. J. Chromatogr. A.

[16] Wimbles R., Melling L.M., Shaw K.J. (2016). Combining electro-osmotic flow and FTA^®^ paper for DNA analysis on microfluidic devices. Micromachines.

[17] Patel M., Kruthiventi S.S.H., Kaushik P. (2021). Polyelectrolyte layer grafting effect on the rotational electroosmotic flow of viscoplastic material. Microfluid. Nanofluid..

[18] Kaushik P., Mondal P.K., Kundu P.K., Wongwises S. (2019). Rotating electroosmotic flow through a polyelectrolyte-grafted microchannel: An analytical solution. Phys. Fluids.

[19] Kumar D., Mehta S.K., Mondal P.K. (2023). Enhanced bio-fluids mixing by the soft polyelectrolyte layer-modulated electroosmotic vortices. Phys. Fluids.

[20] Chen G., Das S. (2015). Electroosmotic transport in polyelectrolyte-grafted nanochannels with pH-dependent charge density. J. Appl. Phys..

[21] Koner P., Bera S., Ohshima H. (2021). Ion-partitioning effects on electrokinetic flow of generalized Maxwell fluids through polyelectrolyte layer-coated nanopore under AC electric field. Colloid Polym. Sci..

[22] Li F., Jian Y., Chang L., Zhao G., Yang L. (2016). Alternating current electroosmotic flow in polyelectrolyte-grafted nanochannel. Colloids Surf. B Biointerfaces.

[23] Wang J., Li F. (2023). Electroosmotic flow and heat transfer through a polyelectrolyte-grafted microchannel with modulated charged surfaces. Int. J. Heat Mass Transf..

[24] Li F., Jian Y. (2019). Solute dispersion generated by alternating current electric field through polyelectrolyte-grafted nanochannel with interfacial slip. Int. J. Heat Mass Transf..

[25] Zhang Y., Zhao G., Xue B., Buren M., Jian Y. (2024). The electrokinetic energy conversion and streaming potential analytical solutions of couple stress nanofluids in the circular polyelectrolyte-grafted nanochannel. Chin. J. Phys..

[26] Hartley N.K., Hayes M.A. (2002). Examination of theoretical models in external voltage control of capillary electrophoresis. Anal. Chem..

[27] Kasicka V., Prusik Z., Sazelova P., Brynda E., Stejskal J. (1999). Capillary zone electrophoresis with electroosmotic flow controlled by external radial electric field. Electrophoresis.

[28] Alizadeh A., Hsu W.-L., Wang M., Daiguji H. (2021). Electroosmotic flow: From microfluidics to nanofluidics. Electrophoresis.

[29] Yang X., Zhao M., Wang S., Xiao Y. (2023). Electro-osmotic flow of Maxwell fluid induced by an alternating electric field in curved rectangular microchannels. Phys. Fluids.

[30] Samaj L. (2022). Electric double layers with modulated surface charge density: Exact 2D results. J. Phys. A Math. Theor..

[31] Alinezhad A., Khatibi M., Ashrafizadeh S.N. (2024). Impact of surface charge density modulation on ion transport in heterogeneous nanochannels. Sci. Rep..

[32] Bian X., Li F., Jian Y. (2021). The Streaming Potential of Fluid through a Microchannel with Modulated Charged Surfaces. Micromachines.

[33] Sun L.-X., Jian Y.-J., Chang L., Zhang H.-Y., Liu Q.-S. (2013). Alternating current electro-osmotic flow of the Maxwell fluids through a circular micro-pipe. J. Mech..

[34] Qing Y., Wang J., Li F. (2024). Electro-osmotic flow and mass transfer through a rough microchannel with a modulated charged surface. Micromachines.

[35] Mandal S., Ghosh U., Bandopadhyay A., Chakraborty S. (2015). Electro-osmosis of superimposed fluids in the presence of modulated charged surfaces in narrow confinements. J. Fluid Mech..

[36] Yang M., Buren M., Chang L., Zhao Y. (2022). Time periodic electroosmotic flow in a pH-regulated parallel-plate nanochannel. Phys. Scr..

[37] Whorton J., Jones T.J., Russell J.K. (2025). Particle settling in a shear-thinning, viscoelastic fluid in the presence of wall effects. Sci. Rep..

[38] Wang K., Shang Y., Wei H. (2011). A finite element penalty method for the linearized viscoelastic Oldroyd fluid motion equations. Comput. Math. Appl..

[39] Sailaja A., Srinivas B., Sreedhar I. (2019). Electroviscous effect of power law fluids in a slit microchannel with asymmetric wall zeta potentials. J. Mech..

[40] Yang P., Lam Y.C., Zhu K.-Q. (2010). Constitutive equation with fractional derivatives for the generalized UCM model. J. Non-Newton. Fluid Mech..

[41] Elhanafy A., Elsaid A., Guaily A. (2020). Numerical investigation of hematocrit variation effect on blood flow in an arterial segment with variable stenosis degree. J. Mol. Liq..

[42] Gayathri K., Shailendhra K. (2014). Pulsatile blood flow in large arteries: Comparative study of Burton’s and McDonald’s models. Appl. Math. Mech..

[43] Bandopadhyay A., Ghosh U., Chakraborty S. (2013). Time periodic electroosmosis of linear viscoelastic liquids over patterned charged surfaces in microfluidic channels. J. Non-Newton. Fluid Mech..

[44] Liu Q.-S., Jian Y.-J., Yang L.-G. (2011). Time periodic electroosmotic flow of the generalized Maxwell fluids between two micro-parallel plates. J. Non-Newton. Fluid Mech..

[45] Zakaria K., Sirwah M.A., Alkharashi S.A. (2011). A two-layer model for superposed electrified Maxwell fluids in presence of heat transfer. Commun. Theor. Phys..

[46] Kuntal Y., Ghiya N., Tiwari A. (2025). Solute dispersion in an electroosmotic flow of Carreau and Newtonian fluids through a tube: Analytical study. Eur. Phys. J. Plus.

[47] Abiev R.S. (2005). A new form of the heat- and mass-transfer and fluid-flow equations. Theor. Found. Chem. Eng..

[48] Jiang J.-Z., Zhang S., Fu X.-L., Liu L., Sun B.-M. (2019). Review of gas-liquid mass transfer enhancement by nanoparticles from macro to microscopic. Heat Mass Transf.

[49] Shen C., Afacan A., Luo J.-L., Ulaganathan J., Klimas S.J. (2016). Solid-liquid mass transfer under flow boiling condition. J. Electrochem. Soc..

[50] Patmonoaji A., Tahta M.A., Tuasikal J.A., She Y., Hu Y., Suekane T. (2023). Dissolution mass transfer of trapped gases in porous media: A correlation of Sherwood, Reynolds, and Schmidt numbers. Int. J. Heat Mass Transf..

[51] Roy D., Bhattacharjee S., De S. (2020). Mass transfer of a neutral solute in polyelectrolyte grafted soft nanochannel with porous wall. Electrophoresis.

[52] Lutsenko V., Yelisieiev V., Sovit Y. (2022). Influence of low-frequency vibrations on mass transfer in pores. IOP Conf. Ser. Earth Environ. Sci..

[53] LeVeque R.J. (2007). Finite Difference Methods for Ordinary and Partial Differential Equations.

